# Study on Clinico-Demographic Characteristics of Orthopaedic Cases in a Tertiary Care Centre: A Descriptive Cross-sectional Study

**DOI:** 10.31729/jnma.5745

**Published:** 2020-12-31

**Authors:** Badri Rijal, Krishna Rana, Srijana KC, Jamuna Lamsal

**Affiliations:** 1 Department of Orthopedics, All Nepal Hospital Private Limited, Samakushi, Kathmandu, Nepal; 2 All Nepal Hospital Private Limited, Samakushi, Kathmandu, Nepal

**Keywords:** *musculoskeletal problems*, *Nepal*, *orthopedics*, *trauma*

## Abstract

**Introduction:**

Orthopedic conditions include a range of condition varying from traumatic injuries, congenital anomalies, chronic back-pain, arthritis, rheumatologic conditions, and other. In Nepal, approximately 2.35 million people are living with musculoskeletal conditions in which 175,000 people are suffering with MSD from non-traumatic causes. The aim of the study is to observe the clinical and demographic pattern of orthopedic problems presenting in the hospital.

**Methods:**

A descriptive cross-sectional study was done in All Nepal Hospital Private Limited in the month of November. The data from the medical record section was retrospectively collected. Sample size of 384 was included and convenience sampling technique was used. The descriptive statistical analysis was done.

**Results:**

Eight hundred forty four cases were included in the study. Implant removal was the main condition for admission in the hospital 105 (12.44%) followed by Forearm Bone Fracture 84 (9.95%) and Cut Injury 64 (7.58%). Most cases presented during the time 6 PM to 12 AM 312 (36.96%).

**Conclusions:**

Most of the orthopedic cases presented in the evening and night time. Implant removal, forearm bone fracture and cut injury form the major bulk of the cases.

## INTRODUCTION

Orthopedic cases comprises from cases with fractures, soft tissue injuries, deformities to congenital bone and joint problems. The burden of musculoskeletal conditions varies from different regions to regions as per their racial, environmental and geographical distribution.^1^ Orthopedic cases are prevalent and they are the top most cause of long-term sever pain and physical disability and also affect the psychosocial status of the affected people as well as their carer.^2^

The statistics of orthopedic cases presenting in the hospital has rapidly rose as the population especially geriatric group rises and there are many challenges to be faced.^6^ Study on clinico-demographic pattern of the orthopedic cases is necessary as it assist in identifying areas for primary prevention. A report given by the Ministry of Health and Nepal Health Research Council has already highlights orthopedic cases as the major cause of the disability countrywide.^6^ This shows that the study on the cases profile of orthopedic cases is necessary to find out the prevalence and pattern of the problem.

The main objective of the study is to study the clinico- demographic pattern of orthopedic cases presenting to the hospital.

## METHODS

A descriptive cross-sectional study was conducted in All Nepal Hospital Private Limited. The study was conducted in the month of November by collecting the data retrospectively from the medical records section. Ethical clearance was taken from the Ethical Review Board of Nepal Health Research Council (Ref No: 1359). Inclusion criteria included the record of cases presenting with orthopedic complaints whereas incomplete record, repeated case data and record with missing diagnoses were excluded from the study. The sample size was calculated as per the given formula:

The sample size of this study is calculated using the formula,

$$\begin{array}{ll} \text{n} & \text{= }{\text{Z}}^{\text{2}}\times \text{p}\times \text{(1}-\text{p)}/{\text{e}}^{\text{2}}\\ & \text{= }{\left(1.96\right)}^{\text{2}}\times 0.5\times (1-0.5)/{(\text{0.05)}}^{\text{2}}\\ & \text{= }384 \end{array}$$

Where,

n = required sample size
Z = 1.96 at 95% Confidence Interval (CI)
p = prevalence, 50%
Margin of error (e) = 5%

Therefore, the calculated sample size was 384. Adding the 10% non-response rate, the sample size that will be taken would be 422. Since the convenience sampling technique will be applied the sample size will be doubled to 844 so as to reduce the possible bias such as information bias.

Data from the medical records were collected and kept in Microsoft Excel. Statistical Package for Social Services (SPSS) Version 16 was used for the statistical analysis. The descriptive statistical analysis was done.

## RESULTS

Among the 844 cases included in the study, musculoskeletal conditions comprising of around 44 conditions were recorded. Among them, implant removal was the main condition for admission in the hospital 105 (12.44%). Forearm Bone Fracture 84 (9.95%) and Cut Injury 64 (7.58%) were the second and third most presenting cases. Similarly, road traffic accident 55 (6.51%), Cellulitis 36 (4.26%), Physical assault 34 (4.02%), tibia and fibula fracture 34 (4.02%) Supracondylar fracture 28 (3.31%), phalanx fracture 28 (3.31%) and fall injury 25 (2.96%) are other common presenting conditions.

**Figure 1:**
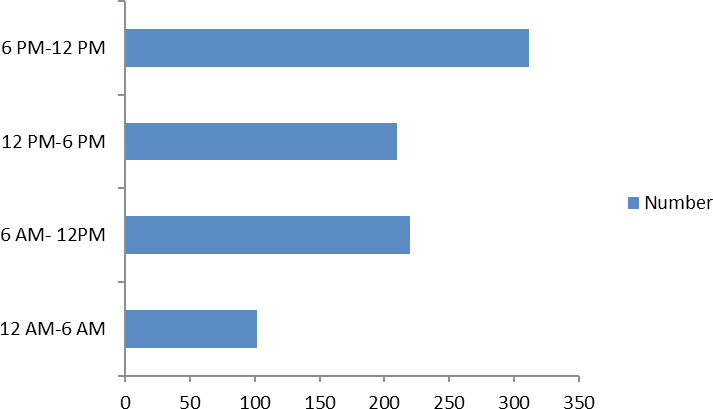
Time of Presentation of Cases

In terms of time of presentation of the cases, most of the cases presented in the time 6 PM-12 AM 312 (36.96%), then the second most influx of cases occurred during 6 AM-12 PM 220 (26.06%) (Figure 1).

Regarding the socio-demographic data of the cases presenting in the hospital, male patients 510 (60.42%) were more than female 334 (39.57%). As per caste, Brahmin 182 (21.56%) were the most followed by Chhetri 171 (20.26%), Newar 142 (17%), Gurung 118 (14%), Tamang 108 (13%), Magar 68 (8%) and others 55 (6%).

**Figure 2:**
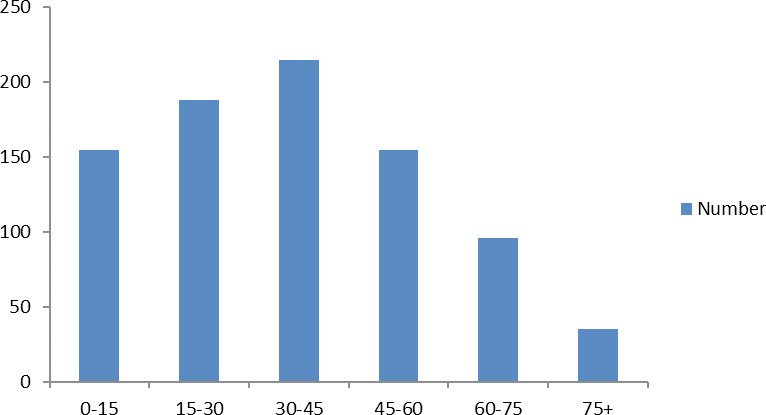
Age-wise Distribution of Cases.

Maximum number of cases presenting came from age group 30-45 215 (25.47%) followed by 15-30 age group 188 (22.27%) (Figure 2). Almost half of the patients came from Bagmati Province 414 (49.02%) accompanied by 242 (28.67%) Gandaki Province.

## DISCUSSION

Orthopedics, as a part of medical science, contribute an immense workload to any kind of tertiary care health centre. Orthopedic cases comprises from cases with fractures, soft tissue injuries, deformities to congenital bone and joint problems. The burden of musculoskeletal conditions varies from different regions to regions as per their racial, environmental and geographical distribution.^1^ A report given by the Ministry of Health and Nepal Health Research Council has already highlightes orthopaedic cases as the major cause of the disability countrywide.^6^

A study done by Syed et al in a tertiary care centre using the 4-year record data showed that most of the cases presenting comes from age 19-50 years and the most frequent diagnoses belong to lower back pain (25.9%), tendinopathies and enthesopathies (18.3%), bone fractures (11%).^7^ Our study showed that the common age group presenting to the hospital came from 30 to 45 years whereas the most commonly admitted cases were implant removal, forearm bone fracture and cut injury. A study done among orthopaedic road traffic accident cases in a tertiary care hospital of Western Nepal revealed most victims as young, unmarried, pedestrians and drunk drivers.^8^

A country-wide cross-sectional survey study done by Chawla et al. showed that approximately 2.35 million people live with musculoskeletal conditions in Nepal.^5^ Similarly, low back pain ranks at the topmost as the cause of disability as per the report submitted by Nepal Health Research Council.^6^ A previous study on orthopaedic conditions in Nepal implicated that falls were the major cause (43%).^5^ However our study shows that fall injury comes at 10^th^ most common presenting condition 25 (2.96%).

A study on Global Burden of Disease from 1990 to 2010 reports that non-traumatic orthopaedic cases account for 6.8% of all disability adjusted life years lost whereas traumatic conditions comprises 11%.^3^ As per World Health Organization, more than 90% of the DALYs lost occur in low- and middle-income countries (LMICs), highlighting the disproportionate burden of orthopaedic cases placed on developing countries.^4^ Our study have highlighted the burden of trauma in orthopedic cases also as 55 (6.51%) cases were found to be admitted secondary to road traffic accident.

This is a single-centre retrospective study, our study may not be truly representative of the epidemiology of orthopedic problems prevalent in the community. The cross-sectional nature of the study, does not allow the establishment of causality.

## CONCLUSIONS

Orthopedic cases comprise mostly of young age and the cases presented in the evening and night time more often. Implant removal, forearm bone fracture and cut injury form the major pattern of musculoskeletal conditions presenting in the hospital. Further study is required with larger sample size and probability sampling method for better scientific findings.
